# Progressing future osteoarthritis treatment toward precision medicine: integrating regenerative medicine, gene therapy and circadian biology

**DOI:** 10.1038/s12276-025-01481-6

**Published:** 2025-06-30

**Authors:** Ee Hyun Kim, Seungho Jeon, Junyoung Park, Ji Hyun Ryu, Ali Mobasheri, Csaba Matta, Eun-Jung Jin

**Affiliations:** 1 Integrated Omics Institute, Wonkwang University, Iksan, Republic of Korea; 2 Department of Biomedical Materials Science, Graduate School of JABA, Wonkwang University, Iksan, Republic of Korea; 3 Research Unit of Health Sciences and Technology, Faculty of Medicine, University of Oulu, Oulu, Finland; 4 Department of Regenerative Medicine, State Research Institute Centre for Innovative Medicine, Vilnius, Lithuania; 5 Department of Joint Surgery, First Affiliated Hospital of Sun Yat-Sen University, Guangzhou, China; 6 Université de Liège, Liège, Belgium; 7 Department of Anatomy, Histology and Embryology, Faculty of Medicine, University of Debrecen, Debrecen, Hungary

**Keywords:** Osteoarthritis, Diagnostic markers

## Abstract

Osteoarthritis (OA) is marked by cartilage degradation, inflammation and varied pain. Traditional treatments such as nonsteroidal anti-inflammatory drugs primarily offer symptom relief without halting disease progression. Advances in regenerative medicine and stem cell and gene therapies, combined with innovative biomaterials such as hydrogels, present new opportunities to target the underlying pathophysiology of OA. This review explores these promising approaches alongside the emerging roles of circadian biology and organelle health in OA pathogenesis and therapy. It highlights the shift toward precision medicine, offering a comprehensive analysis of emerging targeted strategies aimed at improving OA management and patient outcomes.

## Innovative therapies transform osteoarthritis treatment landscape

Osteoarthritis (OA) is a common joint disease that causes pain and stiffness, especially in older adults. Researchers are exploring new therapies to address this issue, here focusing on regenerative medicine, which uses stem cells to repair damaged cartilage. This involves injecting stem cells into joints to promote healing. However, challenges such as cell survival and long-term effectiveness remain. This study also examines gene therapy, which targets specific genes to reduce inflammation and cartilage breakdown. Biomaterials such as hydrogels and nanoparticles are used to deliver these therapies directly to the joint, improving treatment precision. In addition, this research highlights the role of circadian rhythms in OA, suggesting that timing treatments could enhance their effectiveness. These advancements aim to provide more personalized and effective OA treatments. Future research will focus on refining these approaches for better patient outcomes.

This summary was initially drafted using artificial intelligence, then revised and fact-checked by the author.

## Introduction

Osteoarthritis (OA) is a multifactorial joint disease characterized by the progressive breakdown of articular cartilage, inflammation of the synovium and subchondral bone remodeling, leading to joint pain, stiffness and impaired mobility^1^. OA is a leading cause of disability among older adults and the most common form of arthritis worldwide^1^. The prevalence of OA continues to increase, largely driven by aging populations and rising obesity rates, which further contribute to mechanical and biological stress and inflammation in the joint^1^. The etiology of OA is multifactorial and encompasses mechanical, biological, genetic, biochemical and immunometabolic factors that disrupt the balance between cartilage formation and degradation^2^. This intricate interplay complicates OA pathogenesis and underscores the need for innovative and effective treatments that address the root causes of the disease and not just its symptoms.

Conventional pharmacological treatments for OA, such as nonsteroidal anti-inflammatory drugs, corticosteroid injections and nonpharmacological treatments such as exercise and physical therapy, primarily aim to manage pain and reduce inflammation^1^. However, these interventions do not halt the progression of cartilage degradation or reverse joint damage, often leading to the eventual need for more invasive surgical procedures, such as partial or total joint arthroplasty in the most advanced stages of the disease^1^. This unmet medical need has driven a shift in research toward regenerative medicine approaches aimed at repairing damaged tissues and restoring joint function at the cellular and molecular levels.

**Fig. 1:**
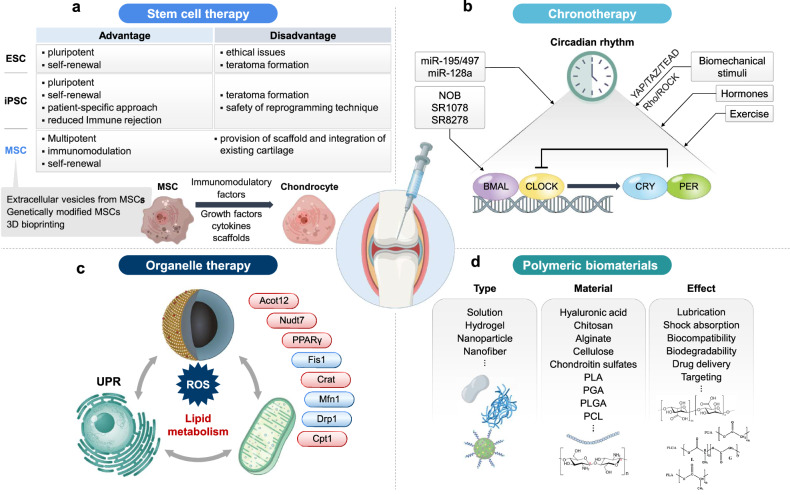
Therapeutic approaches and mechanisms in OA treatment. A schematic illustration integrating multiple therapeutic strategies and their mechanisms, offering a comprehensive view of both current and emerging treatments for OA. **a** Stem cell therapy: the use of various types of stem cells in OA treatment, including ES cells, iPS cells and MSCs. **b** Chronotherapy: the influence of circadian rhythms on treatment efficacy. It details the role of core circadian genes such as BMAL1, CLOCK, CRY and PER, and explores how the timing of treatments can optimize therapeutic outcomes by syncing with these natural biological rhythms. **c** Organelle therapy: the application of organelle therapy in OA by targeting cellular dysfunction and strategies to enhance lipid metabolism and reduce oxidative stress through the activation of specific targets such as PPARγ and the modulation of mitochondrial proteins such as Drp1 and Fis1. **d** Polymeric biomaterials: categorizes various biomaterials such as hydrogels and nanoparticles used in the management of OA.

In recent years, regenerative medicine has emerged as a promising strategy for OA treatment, with mesenchymal stem cells (MSCs) at the forefront owing to their ability to differentiate into chondrocytes, specialized cells that produce the cartilage extracellular matrix (ECM)^3^. In addition, gene therapy has opened new pathways to treat OA at the molecular level by targeting inflammatory and catabolic pathways. Gene therapy has the potential to disrupt key mechanisms driving OA progression by modulating key genes and signaling pathways involved in cartilage degradation and joint inflammation^4^. Targeted delivery systems, such as viral vectors and nanoparticle-based platforms, have been explored to improve the precision and durability of gene therapy. Along with advancements in stem cell and gene therapies, biomaterials have also become integral to the development of OA therapy, providing innovative solutions for delivering drugs, cells and genes directly to the joint^5,6^. Hydrogels, nanoparticles and nanofibers allow for the sustained release and enhanced bioavailability of therapeutic agents, optimizing treatment efficacy while minimizing systemic side effects^5,6^. Circadian biology is another emerging area of OA research, as circadian rhythms influence inflammation and cartilage homeostasis^7,8^. Evidence suggests that disruption of circadian clock genes contributes to OA pathogenesis by disturbing cartilage repair processes and enhancing inflammatory responses^7,8^. Finally, the role of organelles, such as peroxisomes and mitochondria, in OA is gaining attention. These organelles regulate cellular energy metabolism, lipid homeostasis and reactive oxygen species (ROS) detoxification, all of which are crucial for maintaining cartilage health^9,10^. Dysfunction of these organelles has been linked to oxidative stress and metabolic disturbances in OA, indicating that organelle-targeted therapies could provide novel treatment avenues^11,12^. This review provides a comprehensive overview of the current landscape of regenerative therapies for OA, focusing on the potential limitations of MSCs, gene therapy, biomaterials, circadian biology and organelle-targeted treatments (Fig. 1). By exploring these novel approaches, this review aims to underscore the shift toward precision medicine in OA management, emphasizing the need for biologically informed personalized treatment strategies that address the diverse molecular mechanisms underlying OA.

### The current perspectives of regenerative therapy using stem cells in OA

Regenerative OA therapies using stem cells and other cell types have emerged as promising approaches for modifying the disease course and promoting articular cartilage repair (Fig. 1a). Although autologous chondrocytes were initially considered a viable source for cell therapy in cartilage repair, several limitations have emerged. These include adverse changes in chondrocyte phenotype and additional patient discomfort caused by cell collection. Consequently, the field is also focusing more and more on the ‘potency’ of the stem cell sources^13^.

Regenerative therapies for OA rely primarily on the ability of stem cells to engraft and differentiate into chondrocytes. This process involves the administration of stem cells into the joints, which interact with the local microenvironment to stimulate neocartilage production^13^. However, recent studies have highlighted challenges associated with this approach. For instance, injected stem cells may undergo apoptosis, leading to a transient paracrine effect rather than long-term structural improvement^14^. Several types of stem cells with distinct characteristics and advantages are currently being studied for their potential use in OA therapy. Embryonic stem (ES) cells, which are derived from the inner cell mass of blastocysts, are pluripotent and can differentiate into all three germ layers of the embryo. This versatility makes them theoretically attractive for OA therapy. However, ethical concerns and the risk of teratoma formation (tumor growth) limit their clinical application^15^. MSCs are adult stem cells derived from various tissues, including the bone marrow, adipose tissue, Wharton’s jelly and synovium. MSCs possess the capacity to self-renew and differentiate into various cell types, including chondrocytes^3^. MSCs also exhibit immunomodulatory and immunosuppressive properties, potentially mitigating the inflammatory components of OA^16^. Induced pluripotent stem (iPS) cells are adult cells reprogrammed to an ES cell-like state. iPS cells offer a patient-specific approach that potentially reduces the risk of immune rejection^15^. However, similar to ES cells, concerns regarding teratoma formation and safety of reprogramming techniques remain^17^.

Despite these challenges, substantial progress has been made in the development of cell-based regenerative therapies for OA. Preclinical animal models serve as vital platforms for investigating the therapeutic potential of MSCs for OA treatment. These studies focused on elucidating the chondrogenic differentiation and activated signaling pathways in MSCs, particularly within the context of scaffold-free intra-articular (IA) injections. The encouraging results observed in these experimental settings provide a strong rationale for translating MSC therapies into human clinical trials^18^.

The therapeutic effects of MSCs in OA are mediated by a combination of mechanisms. MSCs differentiate into chondrocytes and contribute to cartilage repair and regeneration^19^. Stem cells secrete various immunomodulatory factors that reduce inflammation and promote healing within the joint^20^. They also secrete several growth factors and cytokines that can stimulate the surrounding tissues, promote cartilage matrix synthesis and inhibit degradation^19^. MSCs may also provide a scaffold for new tissue growth and promote integration with the existing cartilage^21^. The trophic activity of MSCs may also be due to the direct interactions and communication between MSCs and chondrocytes through gap junctions^18^. The relative importance of each mechanism may vary depending on the type of stem cell used and the specific stage of the disease. Further research is needed to elucidate the precise mechanisms by which stem cells exert their therapeutic effects on OA.

Several clinical trials are currently ongoing, utilizing stem cells from various sources, including bone marrow and adipose tissue. These trials aimed to determine the efficacy of stem cell therapy in reducing pain and improving joint function in patients with OA, as recently reviewed by Carneiro et al.^18^. Although current pharmacological and nonpharmacological therapies for OA have limited effectiveness, MSC-based therapies hold considerable potential for future advancement. However, this field is still in its early stages and major challenges need to be addressed.

Clinical trials on stem cell therapy for OA have shown mixed results in terms of cartilage repair. MSCs may improve symptoms and function (based on patient-reported outcomes) but lack strong evidence for cartilage regeneration using noninvasive methods such as magnetic resonance imaging^18^. However, clinical trials vary considerably in terms of the study design, cell type, dosage and route of administration. This heterogeneity makes it difficult to draw definitive conclusions regarding efficacy^22^. Most studies had short follow-up periods, making it difficult to assess the long-term durability of treatment effects^18^. The existing meta-analyses have methodological issues and low-quality evidence. Also, the trials did not make any attempt to stratify and phenotype patients^18^. Potential safety concerns associated with stem cell therapy include graft-versus-host reactions to allogeneic cells and teratoma formation in ES cells and iPS cells^15^.

Despite these challenges, several clinical trials on stem cell therapy in arthritic conditions, such as OA, have shown promising results in symptom relief and functional improvement, although there is a lack of clear evidence for complete cartilage regeneration^18^. Although the short-term benefits of MSC therapy are well documented, the long-term efficacy and durability of these treatments remain an area of ongoing research^23^. Larger well-designed studies are needed to confirm these findings and establish the role of stem cell therapy in the routine management of OA. Future trials should follow common guidelines and explore more robust approaches, such as using genetically modified MSCs or combining them with hydrogels, to improve patient outcomes. Furthermore, a great body of research is still needed to optimize treatment protocols, address safety concerns and establish the long-term efficacy of stem cell therapy for OA.

Given that OA is a heterogeneous disease with different clinical phenotypes and molecular endotypes, such as inflammatory, metabolic syndrome, bone and cartilage metabolism and chronic pain phenotypes^24^, patients with different phenotypes may respond differently to MSC therapies. Clinical trials have shown that MSCs can mitigate OA symptoms and improve function in some patients, but the response varies widely^22^. This variability underscores the need for patient stratification to tailor treatments to specific patient phenotypes and endotypes. Developing tools to stratify patients based on clinical and molecular characteristics can help identify those most likely to benefit from specific therapies. Stratifying patients based on OA phenotypes could improve treatment outcomes by matching patients with therapies tailored to their specific disease mechanisms^25,26^.

Future clinical trials should incorporate stratification strategies to enroll patients based on their clinical and molecular profiles. This approach can enhance the efficacy of MSC therapies by ensuring that treatments are applied to the most responsive patient subgroups. Future directions for stem cell-based regenerative therapy for OA include the development of more effective delivery methods, the development of more robust clinical trial designs to ensure the safe and effective application of regenerative medicine in OA treatment and the identification of optimal stem cell types. A recent study showed that Gremlin-1-positive cells, a specific type of chondrogenic progenitor cell, are essential for maintaining healthy cartilage and preventing OA. The discovery of these cells may open new avenues for future research and potential therapies^27^.

Beyond these limitations, there are several promising new directions for stem cell-based regenerative therapies for the treatment of OA. Extracellular vesicles from MSCs may represent a cell-free alternative for injecting trophic factors and may be safer than live MSCs. Genetically modified MSCs may offer a potential approach for enhancing the efficacy of cell therapies and restoring joint health. Three-dimensional bioprinting allows for the precise control of scaffolds to engineer articular cartilage and bone replacements for end-stage OA, potentially avoiding issues with traditional prostheses. Alternatively, scaffolds mimicking natural cartilage, such as gelatin networks with glycosaminoglycans, also show great promise for promoting cartilage regeneration by MSCs^18^.

#### Current regulatory frameworks and their impact on cell-based therapies

The development and implementation of cell-based therapies are strongly influenced by regulatory frameworks that vary globally. These differences can affect the translation of these therapies into clinical practise and the outcomes of clinical trials. Regulatory differences can lead to variability in clinical trial design, approval processes and the interpretation of results. This variability complicates the comparison of outcomes across studies conducted in different regions^28^. In some regions, patients may have access to treatments that are not approved or available elsewhere, which can influence patient outcomes and the perceived efficacy of these therapies.

The EU has a centralized regulatory framework for Advanced Therapy Medicinal Products (ATMPs), which includes cell therapies. The European Medicines Agency oversees the approval process, but individual member states also have roles in implementing regulations, leading to some heterogeneity in legal requirements^29^. The hospital exemption in the EU allows for the use of custom-made ATMPs under specific conditions, which can facilitate access to innovative treatments but also introduces variability in how these therapies are applied^30^. In the USA, the Food and Drug Administration (FDA) regulates cell therapies, with frameworks that are often criticized as outdated and fragmented. The FDA has specific guidance for certain ATMPs, which can lead to different regulatory expectations compared to the EU^31^. In South Korea, the regulatory framework for the use of MSCs in clinical trials is governed by the Ministry of Food and Drug Safety. MSC therapies are classified as cell therapy products under the Act on Advanced Regenerative Medicine and Advanced Biopharmaceuticals, enacted in 2019 and implemented in 2020. This act allows for conditional approval or expedited review for serious or rare diseases where no alternative treatments exist^32^. South Korea adheres to strict medical and ethical standards for stem cell therapies, ensuring patient safety through regulated treatments in accredited facilities^31^.

The lack of harmonization in regulatory frameworks contributes to ‘stem cell tourism’, where patients seek untested or unapproved treatments in countries where stem cell therapies are widely available despite limited regulatory oversight^28^. This phenomenon highlights the need for international cooperation to ensure safety and efficacy standards are met. Addressing these regulatory disparities through harmonization efforts is crucial for ensuring consistent safety and efficacy standards and facilitating global access to innovative treatments.

### Tick tock, the cartilage clock and OA

The circadian clock consists of a molecular network of core clock genes and proteins connected via positive and negative autoregulatory feedback loops that generate ~24 h rhythms in gene expression. The core clock genes include ARNT-like 1 (BMAL1), circadian locomotor output cycle kaput (CLOCK), CRY1,2 (cryptochrome), PER1,2,3 (period circadian regulator), NR1D1,2 (nuclear receptor subfamily 1 group D member 1,2, Rev-ErbA-B) and retinoic acid receptor-related orphan receptors (RORs). BMAL1 and CLOCK activate the expression of CRY1,2 and PER1,2,3, which in turn inhibit the activity of BMAL1 and CLOCK heterodimers, forming a negative feedback loop^33^. These cell-autonomous circadian clocks temporally segregate the activity of key catabolic and anabolic pathways in chondrocytes at different times of the day^34^. The core components of the circadian clock contribute to the homeostasis of articular cartilage^7^.

Circadian rhythms have been implicated in OA pathophysiology (Fig. 1b). The chondrocyte clock weakens with age and is dysregulated due to persistent inflammatory conditions. Circadian rhythm disruption can have important implications for cartilage homeostasis and age-related OA susceptibility owing to the loss of rhythmic balance in cartilage matrix synthesis and catabolic metabolism in the cartilage^8^. Misalignment of physical activity cycles with the optimum circadian phase, determined by local clocks not only in chondrocytes, but also in other musculoskeletal tissues, could result in increased susceptibility to joint injury^8^. Chronic circadian rhythm disturbance can accelerate knee cartilage degeneration in rats accompanied by the activation of the canonical Wnt/β-catenin signaling pathway^35^. Loss of BMAL1 in chondrocytes is associated with the dysregulation of the rhythmic patterns of several genes relevant to cartilage homeostasis and OA^36^, reduced levels of phosphorylated small mother against decapentaplegic (SMAD)2/3 and nuclear factor of activated T cells 2 (NFATC2), and decreased expression of SRY-box transcription factor 9 (SOX9), aggrecan (ACAN) and collagen type II alpha 1 chain (COL2A1)^37^. BMAL1 expression in articular cartilage from the knee joints of patients with OA is negatively correlated with disease severity, and the number of BMAL1-positive chondrocytes is reduced in knee cartilage from aged mice compared with young mice^37^. PER2 is associated with both rheumatoid arthritis and OA, suggesting that altered PER2 expression may be a risk factor for rheumatoid arthritis and that its expression may be affected by inflammation^38^. Genetic disruption of cartilage and intervertebral disc clocks in mice results in an imbalance between anabolic and catabolic processes, leading to progressive degeneration^39^.

Recent research has begun to explore the role of the circadian clock in OA treatment, focusing on the optimization of therapeutic strategies and the potential for chronotherapy^40^. By restricting the timing of drug treatments to maximize efficacy and reduce toxicity, chronotherapy has shown positive results in patients with rheumatoid arthritis, suggesting that it may also be beneficial in OA^38^. There are potential druggable therapeutic targets for the circadian clock in OA treatment. In fact, the ROR agonists nobiletin (NOB) and SR1078, and the Rev-Erb antagonist SR8278 enhanced BMAL1 expression. Furthermore, NOB and SR8278 treatments effectively attenuated the structural destruction of articular cartilage in a surgery-induced OA mouse model^41^. Compounds targeting the molecular components of the circadian clock, such as CRY proteins and Rev-Erbs, could be used in future drug designs^8^.

MicroRNAs (miRNAs) are also involved in regulating the circadian clock in OA. The miR-195/497 cluster influences the circadian rhythm of chondrocytes, resulting in gradual degradation of articular cartilage^38^. Cartilage-specific knockout of miR-128a has a beneficial effect on cartilage integrity in an experimental mouse model of OA by destabilizing the medial meniscus^42^. These results highlight the potential of targeting circadian clock genes to ameliorate age-related changes and susceptibility to OA.

However, targeting the circadian clock in OA treatment presents several challenges. For example, inflammation has been shown to disrupt the expression of circadian clock genes in multiple tissues and cell types, including synovial fibroblasts. It is plausible to hypothesize that increased systemic or local inflammation in the synovial joints, which is frequently observed in aging and OA joints, further contributes to the disruption of the cartilage clock^8^. As OA is a heterogeneous disease, the circadian system may be disrupted in different ways in various OA molecular endotypes, making it challenging to develop a one-size-fits-all therapeutic approach^8^.

Circadian hormones, such as melatonin, thyroid-stimulating hormone and cortisol, are strongly associated with OA. An imbalance in these hormones alters the expression of pro-inflammatory cytokines and cartilage matrix-degrading enzymes, leading to cartilage erosion, synovial inflammation and osteophyte formation^43^. A number of medical conditions, including musculoskeletal and OA pain, have sleep disturbance as a common outcome^44^. The association between circadian rhythm and OA pain can be exploited to develop better therapeutic strategies.

The relationship between the circadian clock and biomechanical stimulation in the treatment of OA is a topic of increasing interest. The importance of mechanical cues in regulating musculoskeletal circadian clock rhythmicity, particularly in the articular cartilage, is now widely accepted^45^. Given that dysregulation of the cartilage clock may contribute to the development of OA, it is essential to understand how mechanical load influences the cartilage clock. Mechanical loading and hyperosmolarity have been identified as daily resetting cues for skeletal circadian clocks^46^, and their disruption may contribute to the risk of developing diseases, such as OA and intervertebral disc degeneration^39^. Mechanical control of the mammalian circadian clock via the Yes-associated protein (YAP)/transcriptional coactivator with PDZ-binding motif (TAZ) and TEA domain (TEAD) signaling pathways may have implications for chondrogenesis^47^. The circadian clock is influenced by stiffness of the extracellular environment via vinculin and the Rho/Rho‐associated coiled‐coil containing kinase (ROCK) pathway, indicating a link between the mechanical properties of the extracellular environment and the circadian clock^48^.

Combining biomechanical stimulation with circadian clock targeting may augment chondrogenesis because mechanical cues have been shown to affect the circadian rhythm in articular cartilage during cartilage formation and in mature chondrocytes^48^. Synchronizing or entraining peripheral clocks through biomechanical stimulation, such as exercise, may help reset circadian rhythms in musculoskeletal tissues, potentially contributing to improved tissue homeostasis and cartilage repair in a noninvasive manner^45^. The emerging knowledge that mechanical stimulation can synchronize stem cell clocks may be beneficial for priming cells for tissue repair, with implications for cartilage tissue engineering and regenerative medicine^45^.

### The translational potential of circadian clock genes in cartilage regeneration

Several studies have implicated the potential of targeting circadian clock genes for therapeutic interventions in OA. Gene therapy may offer a promising means for delivering circadian clock genes to counteract the progression of OA. This approach involves using viral or non-viral vectors to introduce genes into cells, which can then express therapeutic proteins to modulate disease processes^49^. In the context of circadian clock genes, such as *BMAL1*, gene therapy could potentially restore disrupted circadian rhythms in chondrocytes, thereby promoting cartilage repair and reducing inflammation^37^. Studies have shown that overexpressing *BMAL1* can alleviate OA-like changes by reducing the levels of inflammatory cytokines and matrix-degrading enzymes^50^. A study demonstrated that lentiviral vectors encoding the circadian transcription factor *CLOCK* can promote cartilage regeneration and alleviate age-related articular degeneration in mice. This approach not only restored the expression of genes involved in cartilage development but also stabilized heterochromatin, thereby delaying cellular senescence and rejuvenating aged stem cells^51^. Therefore, delivering *BMAL1* or *CLOCK* via gene therapy could be a viable strategy to maintain cartilage health and prevent OA progression.

Recent advances in synthetic biology have led to the development of gene circuits that can preserve circadian rhythms in engineered cartilage tissues, even in the presence of inflammatory cytokines^52^. These circuits can be designed to maintain the expression of clock genes, ensuring that the therapeutic effects of gene therapy are sustained over time. By combining synthetic gene circuits with biomaterial-based delivery systems, it could be possible to create novel therapies that not only restore circadian rhythms, but also protect against inflammation-mediated cartilage degradation. However, further research is needed to fully explore the potential of these approaches and to develop effective treatments that can restore circadian rhythms and promote cartilage regeneration in patients with OA. It should also be noted that while gene therapy holds promise, several challenges need to be addressed, including ensuring targeted delivery, minimizing off-target effects and maintaining long-term gene expression.

### OA gene therapy: key metabolic pathways and hub genes of OA

Maintenance of cartilage homeostasis is crucial for joint health and is tightly regulated by chondrocytes, which are the resident cells of cartilage^53^. Chondrocytes synthesize and maintain the ECM, which is composed of collagen, proteoglycans and other noncollagenous proteins. The balance between anabolic and catabolic activities of chondrocytes is essential for cartilage integrity and function. Several key genes that regulate these processes have been identified, including SOX9, COL2A1, ACAN, matrix metalloproteinase 13 (MMP-13) and A disintegrin and metalloproteinase with thrombospondin motifs 5 (ADAMTS5)^54^. SOX9 is a transcription factor crucial for chondrogenesis and the expression of cartilage-specific ECM components such as COL2A1 and ACAN. MMP-13 and ADAMTS5 are catabolic enzymes that degrade collagen and aggrecan, respectively. Dysregulation of these genes leads to an imbalance between ECM synthesis and degradation, contributing to cartilage destruction in OA, and is mainly mediated by oxidative stress and dysregulated metabolites, especially lipid components^11,12^.

Excessive ROS can cause oxidative damage to cellular components including lipids, proteins and DNA, leading to chondrocyte apoptosis and ECM degradation (Fig. 1c). Dysregulation of this balance can exacerbate oxidative damage and contribute to OA progression^55,56^. Currently, OA is also considered a metabolic disorder with a strong high correlation with obesity and metabolic disease, regardless of controversial results in patients^57^. As a source of energy generation and matrix biosynthesis in cartilage, glucose plays a critical role in maintaining cartilage homeostasis. Dysregulation of glucose metabolism leads to ROS accumulation and cellular damage, ultimately contributing to OA pathology. While the role of glucose metabolism in OA has been extensively studied, this article focuses more on lipid metabolism, another essential aspect of OA pathology. A systematic literature review and meta-analysis revealed that 40% of patients with OA suffered from dyslipidemia, compared with only 8% of patients without OA^58^. In addition, metabolic profiling at different stages of knee OA revealed notable differences in metabolites related to fatty acids, glycerolipids and the tricarboxylic acid cycle^59^. The effect of fatty acids depends on their specific type; saturated fatty acids and 6-polyunsaturated fatty acids increase pro-inflammatory cytokines and apoptosis, whereas n-3 polyunsaturated fatty acid reduces inflammation and cartilage degradation^60^.

The roles of organelles in regulating oxidative stress and lipid metabolism have been well investigated, especially in the mitochondria and endoplasmic reticulum (ER), but scarcely in peroxisomes.

Mitochondrial dysfunction and oxidative stress are well-documented contributors of OA^9^. Mitochondria are the primary source of cellular energy through oxidative phosphorylation and are involved in ROS production. Interleukin-1β (IL-1β) stimulates mitochondrial fission through Drp1 and inhibited fusion through Mitofusin 1 (Mfn1) in chondrocytes, resulting in mitochondrial dysfunction, increased ROS and dysregulated apoptotic pathways^61^. Inhibition of autophagy caused by Parkin deficiency results in the accumulation of damaged mitochondria and enhances oxidative stress in OA chondrocytes^62^. Mitochondria regulate lipid and glucose metabolism. Carnitine palmitoyltransferase 1 (CPT1) is a transporter of long-chain fatty acids to mitochondria for β-oxidation and AMP-activated protein kinase (AMPK)/acetyl-CoA carboxylase (ACC)/CPT1 signaling reduced lipid accumulation and mitochondrial dysfunction via l-carnitine in synoviocytes^63^.

ER stress and the unfolded protein response (UPR) have also been implicated in OA pathogenesis^64^. ER is responsible for protein folding, lipid synthesis and calcium storage. Disruption of ER function can lead to the accumulation of misfolded proteins and activation of UPR, which aims to restore ER homeostasis. Chronic ER stress induces apoptosis and inflammatory responses, contributing to cartilage degeneration^64^. Palmitate treatment increased ER stress and apoptosis in human articular chondrocytes^65^ and high-fat diet-induced OA lesions were ameliorated through a selective inhibitor of ER stress, 4-phenyl-butyric acid^66^.

In addition to the well-established roles of these factors, recent studies have highlighted the importance of peroxisomal genes and their protein products in cartilage homeostasis and OA pathogenesis. Peroxisomes are small membrane-bound organelles that play a critical role in lipid metabolism, ROS detoxification and regulation of cellular redox status^67–69^. Peroxisomes contain antioxidant enzymes, such as catalase, which decompose hydrogen peroxide into water and oxygen, thus mitigating oxidative stress. Dysfunctions in the peroxisomal pathways have been implicated in various metabolic disorders and are linked to OA. Analysis of fatty acid profiles of synovial fluids from patients with OA revealed that nervonic acid and tetracosadienoic acid, which are very long-chain fatty acids preferentially beta-oxidized in peroxisomes, were markedly increased^70^. Accumulation of both long-chain fatty acids and very-long-chain fatty acids in OA chondrocytes^71^ and high expression levels of peroxisomal biogenesis factor (PEX)14, catalase (CAT) and ATP Binding Cassette Subfamily D Member (ABCD) 3 in hypertrophic chondrocytes also imply an important role of peroxisomes in cartilage homeostasis^72^.

Nudix hydrolase 7 (nudt7) and acyl-CoA thioesterase 12 (acot12) are peroxisomal genes and proteins that have garnered attention for their roles in cartilage health^73,74^. Nudt7 is a member of the Nudix hydrolase family that specifically hydrolyzes coenzyme A (CoA) derivatives, which are key intermediates in lipid metabolism. By regulating CoA levels, Nudt7 modulates peroxisomal β-oxidation and lipid homeostasis. A recent study showed that Nudt7 expression was altered in OA cartilage, suggesting a potential link between peroxisomal function and cartilage degradation^73^. Acot12 is another peroxisomal enzyme that hydrolyzes acyl-CoA thioesters into free fatty acids and CoA. This activity is crucial for maintaining lipid homeostasis and preventing accumulation of toxic lipid intermediates. The dysregulation of Acot12 can lead to lipid imbalance and oxidative stress, thereby contributing to cartilage damage and OA progression^74^. The role of peroxisomal biogenesis and function in chondrocyte homeostasis is further supported by studies of peroxisome proliferator-activated receptors (PPARs), a group of nuclear receptor proteins that regulate the expression of genes involved in lipid metabolism, inflammation and cellular differentiation^75^. PPARγ, in particular, has been shown to play a protective role in cartilage by promoting the expression of anabolic genes and inhibiting inflammatory and catabolic pathways^76^. Activation of PPARγ has been proposed as a therapeutic strategy for OA, given its ability to modulate lipid metabolism and reduce inflammation^77^. The relationship between PPARγ signaling, peroxisomal function and cartilage homeostasis underscores the interconnected nature of these pathways.

The interplay between peroxisomes and other cellular organelles, such as mitochondria and ER, is critical for maintaining cellular homeostasis^10^. The crosstalk between these organelles is essential for cellular energy metabolism and redox balance, which are crucial for chondrocyte function and cartilage health. The interplay between mitochondrial ROS production and peroxisomal ROS detoxification is crucial for maintaining cellular redox balance^78^. Peroxisomes can influence mitochondrial function through the regulation of peroxisomal β-oxidation and the generation of signaling molecules such as ROS and lipid intermediates^78,79^. Fission1 (Fis1), which is essential for mitochondrial fission, is decreased in OA chondrocytes, thereby contributing to peroxisomal and mitochondrial dysfunction^80^. Carnitine acetyltransferase (Crat), which is crucial for the transport of acetyl-CoA from peroxisomes to mitochondria, has been reported to be decreased in patients with OA, leading to lipid accumulation, apoptosis and decreased catalase activity^71^. The interaction between ER stress and peroxisomal dysfunction is an emerging area of interest because both organelles are involved in lipid metabolism and ROS regulation^10^. Understanding the molecular mechanisms underlying crosstalk between these organelles may reveal novel therapeutic targets for OA.

### Polymeric biomaterials for the treatment of OA

Recently, there has been growing interest in the use of polymeric biomaterials, such as natural polymers (for example, hyaluronic acid (HA), chitosan and alginate) and synthetic polymers (polylactic acid (PLA), polylactic-co-glycolic acid (PLGA) and polycaprolactone (PCL)) for the treatment of OA^5,6,8,10^ (Fig. 1d). These biomaterials can be utilized to effectively deliver therapeutic drugs or genes using drug-delivery carriers, repair cartilage using tissue-engineered scaffolds or enhance chondroprotection by the IA injection of a viscosupplement^5,6,81^. Polymers without any modifications or additives can contribute to the management of symptoms and delay OA progression. For instance, HA injections provide lubrication and shock absorption in the knee joints by reducing friction^82^. However, the applications in OA treatments are limited owing to the intrinsic properties of polymers. Various chemical modifications of polymers and preparation of polymer composite materials have been reported to overcome the limitations of using polymers alone. Therefore, it is highly desirable to understand the general aspects of polymeric biomaterials, polymer modifications and polymer composites for the treatment such as drug delivery, cartilage tissue regeneration and next-generation viscosupplements.

Polymeric biomaterials can be processed into various physical states, such as solutions, hydrogels, patches/sponges, nanofibers and micro/nanoparticles. One of the most commonly used physical states of polymeric biomaterials is a three-dimensional, hydrophilic and crosslinked polymer network that absorbs a large amount of water^83^. Hydrogels are used in the context of OA treatment because of their ability to mimic the natural ECM of cartilage, provide a supportive environment for cartilage cells and promote tissue regeneration^84^. For instance, hydrogels can be injected into the joint cavity to enhance the viscoelastic properties of the synovial fluid in a procedure known as viscosupplementation^85^. Nanoparticles are also commonly used for the treatment of OA^86^. Nanoparticles are effective for delivering therapeutic drugs because of their enhanced cellular uptake. The controlled release profiles of therapeutic drugs from nano/microparticles maintain the therapeutic window in the target tissue, resulting in reduced inflammation and promotion of cartilage tissue regeneration. In addition, the modification of nanoparticles with targeting ligands provides localization and immobilization of drug release to improve therapeutic effectiveness^87^. Nanofibers have a high surface-area-to-volume ratio, flexibility and the ability to form porous structures that can be used to deliver drugs and support tissue regeneration in the treatment of OA^88^. Similar to other physical states of hydrogels, nanoparticles, patches and nanofibers can encapsulate therapeutic agents and gradually release the drugs over time. Thus, the physical state of the polymeric biomaterials is important for the treatment of OA.

HA, a negatively charged, naturally occurring polysaccharide composed of repeated units of *N*-acetylglucosamine and glucuronic acid, is a natural polymer widely used not only for the treatment of OA, but also in various biomedical applications, such as wound dressings, drug-delivery depots and tissue-engineering scaffolds, because of its biocompatibility and biodegradability^89,90^. HA and its derivatives are used for OA treatment as drug-delivery carriers, tissue-engineering scaffolds and chondroprotective viscosupplements. Various HA derivatives have been synthesized as drug-delivery systems. Methotrexate-, alendronate-, diclofenac-, triamcinolone acetonide- and dexamethasone-conjugated HA have been synthesized as prodrugs^91–94^. Articular injection of alendronate-conjugated HA reduced MMP-13, MMP-3, interleukin-6, vascular endothelial growth factor and caspase-3 (refs. ^92,95^). In addition, tyramine-, methacrylate- and fibrin-conjugated HA and crosslinked HA have been used for drug delivery^96–99^. The epigallocatechin-3-gallate (EGCG)-containing HA–tyramine hydrogels induce chondrogenic regeneration in vitro and minimize cartilage loss in a surgically induced OA model^96^. HA and its composites have been used for cellular and gene delivery^100,101^. The mixture of HA and MSCs prevented cartilage loss and surface abrasion with enhanced histological scores and cartilage content^100^. In addition, chondrocyte and ADAMTS-targeting gapmer-loaded HA and chitosan composite hydrogels efficiently knocked down ADAMTS5 with a sustained release profile^98^. Without any drugs and cells, HA- and lactose-modified chitosan mixtures also counteract oxidative stress caused by ROS and restore the transcription of IL-1β, TNF, Gal-1, MMP-3 and MMP-13 (ref. ^102^). In addition, HA provides chondroprotection through lubrication and shock absorption, as previously mentioned. Direct injection of high-molecular weight HA into the synovium has been reported to restore the viscoelastic properties of the synovial fluid, resulting in pain relief, improved joint function and enhanced joint mobility^82^. For the use of HA in OA treatments, modification or crosslinking of HA is often utilized to prolong its stability because of its rapid degradation profiles in vivo^103–107^. Carnosine-, dopamine- and ureidopyrimidinone-conjugated HA were synthesized for chondroprotection^103–105^. For example, IA injection of ureidopyrimidinone-conjugated HA showed improved lubrication effects with prolonged stability compared with unmodified HA^105^. HA derivatives and their composites are useful for treating OA because of their biocompatibility, biodegradability and enhanced functional properties.

Chitosan is a positively charged, naturally occurring polysaccharide that is a copolymer of d-glucosamine and *N*-acetyl-d-glucosamine units. Chitosan is useful in OA treatment because it promotes cartilage regeneration and reduces inflammation^108^. IA injection of chitosan oligosaccharide solution without additives upregulated the osteoprotegerin/ligand for the receptor RANK (RANKL) ratio and downregulated the RANKL/receptor activator of NF-κB (RANK) ratio^109,110^. In addition, oral administration of chitosan oligosaccharides inhibited Inducible nitric oxide synthase (iNOS) and cyclooxygenase-2 (COX-2) expression and suppressed synovial inflammation both in vitro and in vivo^111^. In addition, chitosan-based nanoparticles/microspheres have been used for drug (that is, ginsenoside compound K, ketoprofen and sinomenium), protein (that is, anti-inflammatory peptides and superoxide dismutase) and gene delivery (plasmid DNA)^112–117^. Chitosan-based materials can also be used in chondroprotection^102,117^. *N*-Carboymethyl chitosan, aldehyde-modified HA, adipic acid dihydrazide composite hydrogels inhibit the inflammatory cytokines (TNF, IL-1β, IL-6 and IL-17), resulting in chondroprotection against cartilage destruction^118^. In addition, the lactose-modified chitosan and boric acid composite hydrogels showed ROS scavenging activities, which are involved in OA pathology^102^. Thus, chitosan derivatives and their composites are excellent candidates for OA treatment.

Other naturally occurring polysaccharides widely used in the treatment of OA include alginate, cellulose and chondroitin sulfate^119–121^. IA injection of alginate inhibited OA progression, decreased cartilage degradation and reduced potential cytokine stimulation, with an improvement in the friction coefficient^122,123^. In addition, glucosamine sulfate-, cholinium caffeate- and betamethasone dipropionate-containing alginate-based nanoparticles, beads and microcapsules have been used for the delivery of bioactive molecules^124–126^. Alginate composite hydrogels (that is, oxidized alginate/gelatin and alginate/collagen) have also been used as tissue-engineering scaffolds^127,128^. The self-crosslinked oxidized alginate and gelatin hydrogels, in the presence of borax, reduced inflammatory and oxidative stress with enhanced GAG deposition and the formation of hyaline cartilage^127^. In addition to alginate, cellulose derivatives are also used in OA treatment^129–131^. Resveratrol-loaded cellulose-based aerogels inhibit the expression of COX-2 and MMP-13, and suppress the levels of IL-6 and TNF inflammatory factors^131^. The injection of autologous nasal chondrocytes associated with hydroxypropyl methylcellulose hydrogels is effective for the formation of repaired tissue with a histological organization similar to that of healthy articular cartilage^130^. Chondroitin sulfate is a glycosaminoglycan commonly found in various connective tissues, including cartilage. Chondroitin sulfate has enormous potential for OA treatment because it inhibits extracellular proteases and stimulates proteoglycan production with anti-inflammatory properties^132,133^. For instance, aldehyde-containing methacrylated chondroitin sulfate exhibits excellent adhesion to cartilage tissue, overcoming the challenges of adhesion and integration to cartilage for biological cartilage repair^132^. Chondroitin sulfate can also be used to manage OA. IA injection of a combination of chondroitin sulfate and HA reduced pain, clicking and limited mouth opening in a randomized clinical trial^134^.

Other synthetic polymers used to treat OA are PLA, polyglycolic acid (PGA), PLGA and PCL, among many others^6,135,136^. The IA injection of etoricoxib-loaded PLA/chitosan nanoparticles exhibit enhanced ALP activity and increased calcium ion deposition and binding^135^. In addition, chondroitin sulfate-loaded PLGA microspheres readily controlled the multiple burst release of chondroitin sulfate by regulating the size of the microspheres for the treatment of OA^137^. Nanoparticle-containing polymeric networks are often used to retain viscosity. The combination of HA and PLGA nanoparticles showed excellent anti-inflammatory activity compared with HA solution alone^136^. Thus, the use of nanoparticles or combinations of nanoparticles and other physical states may improve the therapeutic effects in cartilage tissue regeneration. PCL is a biodegradable polyester with a low degradation rate and excellent biocompatibility. Lignin-grafted PCL nanofibers and etoricoxib-encapsulated PCL microparticles in chitosan hydrogels have also been developed for OA treatment^138,139^.

## Concluding remarks and perspectives

This narrative review highlights the evolving landscape of OA management, particularly focusing on regenerative therapies, advanced biomaterials, the integration of circadian biology and organelle-targeted therapies into treatment strategies. Regenerative medicine, particularly stem cell therapy, offers hope of modifying the course of OA. Challenges associated with stem cell therapy include variability in study design, types of stem cells used, dosages and administration routes. Moreover, the transient nature of the observed therapeutic effects raises questions about the long-term efficacy and safety of such interventions^18^. Future research must focus on standardizing protocols for stem cell therapy, exploring genetically modified MSCs to enhance their therapeutic potential and investigating alternative sources such as extracellular vesicles that may provide similar benefits without the risks associated with live-cell therapies.

In addition to cellular therapies, recent advancements in biomaterials have shown promise for enhancing drug delivery and tissue repair. The development of responsive polymers and hybrid composites tailored for IA delivery can substantially improve the bioavailability of therapeutic agents. These innovations not only facilitate localized treatment, but also minimize the systemic side effects associated with traditional pharmacological approaches. The integration of biomaterials with stem cell therapy could potentially enhance the engraftment and survival of transplanted cells, while providing a supportive microenvironment for cartilage regeneration^140^. Future studies should aim to optimize these combinations to maximize the therapeutic outcomes.

The exploration of circadian biology in OA management represents a novel approach that could revolutionize treatment strategies. Chronotherapy has already shown promise in other inflammatory conditions such as rheumatoid arthritis; thus, similar strategies could be adapted for OA management. Targeting the circadian clock genes and their associated pathways may provide new therapeutic avenues to mitigate age-related changes in cartilage metabolism and enhance treatment efficacy^141^. However, challenges remain in understanding how individual variabilities in circadian rhythms affect OA pathology and treatment responses.

Emerging evidence suggests that organelles involved in ROS regulation and lipid metabolism are promising therapeutic targets for OA. Enhancing the function of key organelles, particularly mitochondria and the ER, has shown potential in mitigating OA pathology. However, the role of peroxisomes in OA remains largely underexplored, despite their known association with lipid metabolism and ROS regulation. A comprehensive investigation into the interplay between peroxisomes and other organelles is essential for developing effective OA treatments.

Although the current therapies for knee OA offer limited efficacy in halting disease progression or restoring joint function, emerging strategies involving regenerative medicine, advanced biomaterials, circadian biology and organelle biology offer promising new direction. Given the diverse underlying mechanism of OA among patients, personalized approaches that target dysregulated cellular mechanisms with appropriate biomarkers are likely to be more effective and safer. MSC therapy, gene therapy targeting circadian clock mechanisms or metabolite-regulating organelles and optimized biomaterial application could provide substantial leverage for advancing personalized medicine. Successfully overcoming existing regulatory and safety challenges at the clinical stage will be crucial. Further research is essential to address these challenges and translate innovative strategies into clinically effective treatments. As we move forward, the integration of multidisciplinary and multimodal approaches will be crucial in reshaping future OA management paradigms toward more effective patient-centered care.
